# Conflation of Short Identity-by-Descent Segments Bias Their Inferred Length Distribution

**DOI:** 10.1534/g3.116.027581

**Published:** 2016-03-01

**Authors:** Charleston W. K. Chiang, Peter Ralph, John Novembre

**Affiliations:** * Department of Ecology and Evolutionary Biology, University of California, Los Angeles, California 90095; † Department of Molecular and Computational Biology, University of Southern California, Los Angeles, California 90089; ‡ Department of Human Genetics, University of Chicago, Illinois 60637

**Keywords:** identity-by-descent, coalescent, human genetics

## Abstract

Identity-by-descent (IBD) is a fundamental concept in genetics with many applications. In a common definition, two haplotypes are said to share an IBD segment if that segment is inherited from a recent shared common ancestor without intervening recombination. Segments several cM long can be efficiently detected by a number of algorithms using high-density SNP array data from a population sample, and there are currently efforts to detect shorter segments from sequencing. Here, we study a problem of identifiability: because existing approaches detect IBD based on contiguous segments of identity-by-state, inferred long segments of IBD may arise from the conflation of smaller, nearby IBD segments. We quantified this effect using coalescent simulations, finding that significant proportions of inferred segments 1–2 cM long are results of conflations of two or more shorter segments, each at least 0.2 cM or longer, under demographic scenarios typical for modern humans for all programs tested. The impact of such conflation is much smaller for longer (> 2 cM) segments. This biases the inferred IBD segment length distribution, and so can affect downstream inferences that depend on the assumption that each segment of IBD derives from a single common ancestor. As an example, we present and analyze an estimator of the *de novo* mutation rate using IBD segments, and demonstrate that unmodeled conflation leads to underestimates of the ages of the common ancestors on these segments, and hence a significant overestimate of the mutation rate. Understanding the conflation effect in detail will make its correction in future methods more tractable.

In the present study, we consider a genomic region to be shared identically by descent (IBD) between a pair of individuals if the region was coinherited from a common ancestor without any intervening recombination. This and related concepts of coinheritance and IBD have been useful in numerous applications. For example, identifying shared haplotypes forms the basis of several imputation (Gusev *et al.* 2012) and phasing (Kong *et al.* 2008; S. R. Browning and B. L. Browning 2011) methods; the frequency of IBD within and between cohorts allows the detection of natural selection and trait-associated loci (Purcell *et al.* 2007; Albrechtsen *et al.* 2010; Gusev *et al.* 2011; Han and Abney 2013); and contrasting the number and length of IBD segments enables inferences of past demographic histories (Palamara *et al.* 2012; Ralph and Coop 2013). The concept of IBD has also been used in recent efforts to estimate key human genetic parameters such as the mutation rate (Campbell *et al.* 2012, Palamara *et al.* 2015) and trait heritability (Zuk *et al.* 2012).

Traditionally, the concept of regions or segments sharing IBD has been defined with respect to a set of founder individuals, such as in a pedigree (Thompson 2013). More recently, attention has been paid to IBD segments among pairs of individuals in populations without known pedigree. Since all homologous regions trace their ancestry to a single common ancestor, and since the definition we adopted is with respect to recombination, not mutation, each individual is IBD with every other individual at every position in the genome. In random samples from unstructured populations, most of these IBD segments shared by a pair of individuals are short relative to the spacing of polymorphic sites (Chapman and Thompson 2003; Powell *et al.* 2010; Thompson 2013). For this reason, only particularly long and hence recent segments of IBD can be reliably identified. In practice, the segments studied are the subset of all IBD segments that exceed a particular length threshold which, on the basis of estimated error rates in humans, is often chosen to be between 1–2 cM (B. L. Browning and S. R. Browning 2011). However, this choice could depend on the behavior of the specific algorithm one uses and the research question one seeks to answer.

A number of computer programs exist to detect IBD segments using high-density array data from population samples. Beagle IBD (S. R. Browning and B. L. Browning 2010), RELATE (Albrechtsen *et al.* 2009), IBDLD (Han and Abney 2011), and PLINK (Purcell *et al.* 2007) detect IBD segments based on a probabilistic hidden Markov model for IBD status to determine the posterior probability of IBD at a genomic location. Probabilistic models tend to be computationally intensive (B. L. Browning and S. R. Browning 2013b), thus it is often unfeasible to apply them to large population datasets. On the other hand, programs like GERMLINE (Gusev *et al.* 2009), Beagle fastIBD (B. L. Browning and S. R. Browning 2011), and Refined IBD (B. L. Browning and S. R. Browning 2013b) adopt a dictionary approach to detect IBD, followed by varying degrees of probabilistic assessment of IBD segments to improve accuracies. These dictionary approaches are scalable to large sample datasets. These methods identify IBD segments by looking for long stretches of near-identical sequences (identity-by-state, or IBS). This is unambiguously useful; but to make use of these data in a model-based framework, some applications then assume that such segments, if long enough, have been uninterrupted by intervening recombination, and thus coinherited from a single common ancestor.

This assumption, as we will examine in the current manuscript, does not always hold. It is clear that long segments of IBS could be due to the conflation of two or more shorter IBD segments, especially using diploid data, where the two IBD segments could be shared between different pairs of haplotypes. This error in the estimated length will in turn lead to errors in the estimated ages of common ancestors and bias downstream inferences. For example, if unmodeled, conflated segments would be inferred to derive from an erroneously recent common ancestor, thus biasing estimates of population size and migration rate. Similarly, and as we will show, an approach to estimate the mutation rate using the length of IBD segments would erroneously assign younger ages to conflations of older segments and thus overestimate the mutation rate.

Conflation of neighboring IBD segments is one mechanism leading to misestimation in IBD calling. B.L. Browning and S. R. Browning (2013b) have previously examined the problem of overestimation of IBD segment lengths in IBD calling algorithms, implicitly grouping misestimation due to conflation with other factors such as genotyping errors. To help distinguish sources of error, we refer to “conflation” as the conflation of two neighboring long segments, resulting in a large error in estimated length, and “minor endpoint errors” as any other error that leads to a small error in estimated length. From the standpoint of the calling algorithm, at the end of a nonconflated segment the statistical evidence of IBD should decrease as sequence mismatches accumulate in regions where two individuals are very distantly related; eventually the evidence of IBD falls below the necessary threshold and the called segment is terminated. This is what we consider a minor endpoint error, which could be alleviated by simply removing ends of called IBD segments or more sophisticated statistical modeling in subsequent analysis. On the other hand, at the end of a conflated segment, the statistical evidence for IBD actually increases as the algorithm considers markers in the neighboring IBD segment, and continues until the end of the second segment. Thus, the problem of conflation of segments that we study here is one that will not disappear with increasing marker spacing or accuracy, as it is driven by the rate at which true segments of an appreciable length are spatially clustered in the genome. Indeed, it is this conflation that may have pushed the summed length of IBD in a region over the length threshold necessary for detection. Furthermore, this kind of error, as we show here, is more problematic for shorter segments near the calling threshold and in diploid organisms with imperfect phasing. Though researchers in the field recognize that IBD segment distributions are biased and often compensate for these effects via simulation or numerical procedures, our aim is to explicitly highlight and characterize this potential mechanism for the biased length distribution of IBD segments.

To characterize the problem, we used coalescent simulations where we know the precise ancestral recombination graph [ARG (Hudson 1991; Griffiths and Marjoram 1996)] of the simulated sample. At all positions of a simulated sequence, the ARG relates individual haplotypes via a local genealogical tree. As in B. L. Browning and S. R. Browning (2013b), we identify true IBD segments between two individuals as those stretches of contiguous sequence on which the time back to the most recent common ancestor between the two does not change (Figure 1).

**Figure 1:**
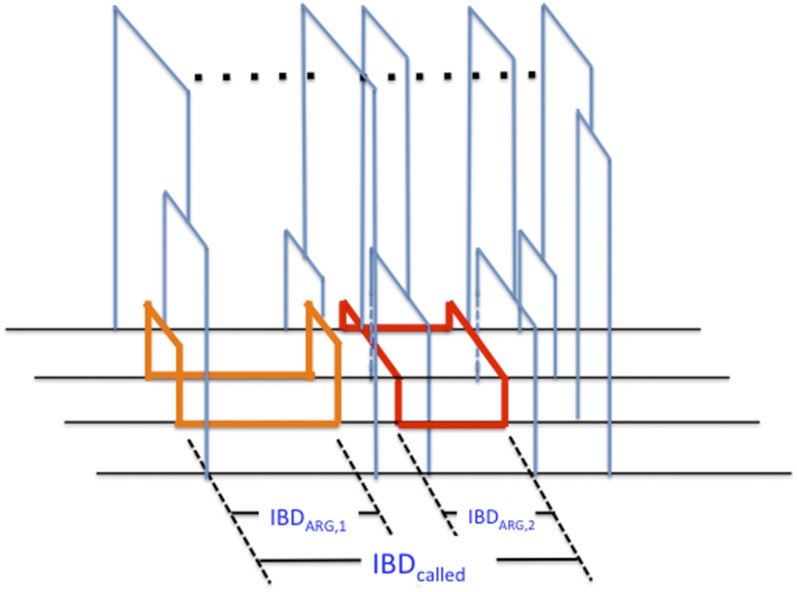
A schematic relating IBD_ARG_ and IBD_called_ segments. The cartoon shows the alignment of four haplotypes belonging to two diploid individuals. Across the region multiple ARGs exist to relate the four haplotypes in a tree. Across the first IBD_ARG_ region (orange, IBD_ARG,1_), the two middle haplotypes have recent, unchanging, local graphs for the entire region. Across the second IBD_ARG_ region (red, IBD_ARG,2_), two different haplotypes have recent, unchanging, local graphs for the entire region. The two IBD_ARG_ segments happens to occur near each other such that the entire region may be detected by algorithms based on long stretches of sequence similarity (IBD_called_ segment). ARG, ancestral recombination graph; IBD, identity-by-descent.

We then simulated genotype data, from which we inferred IBD segments using published algorithms, and contrast the algorithm-detected IBD segments to the true IBD segments determined from the ARGs. We show that, under various demographic models appropriate for modern humans, a significant proportion of the detected IBD segments 1 cM or longer (predominately due to those between 1–2 cM long) are composed of at least two subsegments. As an example of how the conflation can lead to practical problems, we analyze the behavior of a potential estimator of the *de novo* mutation rate using IBD segments. We observed accurate estimates of the input mutation rate when true IBD segments were used, but overestimates of the mutation rate by ∼10–40% using inferred IBD segments, depending on the amount of conflation in the dataset. Our analysis focused on using one of the most recent IBD calling algorithms for array data, Refined IBD (B. L. Browning and S. R. Browning 2013b), but we also tested a number of other algorithms designed to detect IBD segments from array data, and demonstrate that the conflation problem is faced by all algorithms tested.

## Methods

### Coalescent simulations

We simulated five 20 Mb regions of 2000 haplotypes each using MaCS (Chen *et al.* 2009). To mimic genetic data of actual populations, we simulated under a demographic model previously estimated for a European population (Nelson *et al.* 2012). Specifically, we modeled a population with constant size: 12,500 diploid individuals up until 17,000 generations ago, 24,500 between 3500–17,000 generations ago, 7700 between 368–3500 generations ago, and exponentially expanding at a rate of 0.017 per generation (gen)to a present day size of 4 × 10^6^. We assumed constant mutation rate (1.2 × 10^−8^ per bp per gen) and recombination rate (1 × 10^−8^ per bp per gen). The MaCS command used is given in the *Appendix*.

To generate phased sequence data from the MaCs output, we paired the genotypes from two randomly chosen simulated haplotypes. We introduced no missing data or genotyping errors. For our parameter settings, MaCS produced an average of 255,587 (ranged from 254,984 to 256,125) variants per simulated regions. To generate genome-wide (unphased) array data, we down-sampled the sequencing data to match the marker density and allele frequency spectrum typical of array data. The marker density was 5800 markers per 20 Mb region, which extrapolates to approximately 715,000 markers across the entire set of autosomes for which recombination rate estimates are available (approximately 2450 Mb). This density is typical of a genome-wide 1 M array after quality controls and filtering on common variants. The minor allele frequency spectrum was empirically matched to that found on an array, based on the array data from European individuals (*i.e.*, approximately a flat distribution for variants > 5% frequency). Markers with minor allele frequency < 5% were dropped from the array data. In total, an average of 5833 (range 5721–5944) variants were retained for the simulated array data for each simulated region. We next used simulated array data to call IBD segments with a number of available IBD calling algorithms, and then used the simulated sequencing data to infer mutation rate (below).

In addition to the plausible demographic model from Nelson *et al.* (2012), we also tested a number of other demographic trajectories: the European and African demography from Tennessen *et al.* (2012), the same European model but without the second, rapid, exponential growth phase, as well as a constant size population of 100,000 individuals. Detailed descriptions of the demography and MaCS commands used for these scenarios can be found in the *Appendix*.

### Detecting IBD_called_ segments

The main algorithm of IBD detection tested here is Beagle’s Refined IBD (B. L. Browning and S. R. Browning 2013b), though we also tested a number of other algorithms designed to detect IBD segments from array data, namely fastIBD (B. L. Browning and S. R. Browning 2011), GERMLINE (Gusev *et al.* 2009), IBDLD (Han and Abney 2011), and PLINK (Purcell *et al.* 2007).

We followed the recommendations of the authors of Refined IBD/Beagle and called IBD segments in our simulated dataset using Beagle 4.0 (version 1399) with *ibdtrim* and *overlap* parameters set to typical marker density in 0.15 cM and 1.5 cM windows in the dataset, respectively. We used the default LOD score of 3. Only segments greater than 1 cM were retained for analysis. To avoid artifacts due to the edges of simulated regions, we also removed segments that overlapped the boundary of the simulated region. In total, we were left with 5459 IBD segments greater than 1 cM in length across the five simulated regions (between 1036–1157 segments per region).

For comparison with IBD segments called by other algorithms, we also used GERMLINE (v.1.5.1), fastIBD (v.3.3.2), and IBDLD (v.3.004.4) to call IBD segments (Gusev *et al.* 2009; B. L. Browning and S. R. Browning 2011; Han and Abney 2013). We applied each method to only a single simulated region to reduce the amount of computation.

To estimate IBD segments using GERMLINE, we first subjected the simulated data to 10 iterations of phasing by Beagle. Following previous performance comparisons of GERMLINE to Refined IBD, we required the minimum length of the segment to be 1 Mb (–min_m = 1), and seed size for exact matching to be 32 (–bit 32). However, we set –err_hom and –err_het to be 0, as we introduced no genotyping errors in our simulation and higher values could spuriously lead to more apparent conflations. We also used the haploid mode (–h_extend), which allows GERMLINE to utilize the haplotype phase information, and should improve performance (B. L. Browning and S. R. Browning 2013b). Default parameters were otherwise used. Increasing the value of bit parameter decreased the number of segments detected but produced otherwise qualitatively similar results (data not shown). We also used –haploid on the computationally phased data and obtained qualitatively similar results (data not shown). We did not test GERMLINE in haploid mode with perfectly phased data (from simulation) as it is unrealistic in practice. In total, we detected 786 IBD segments greater than 1 cM from one simulated region.

Default settings were used to estimate IBD segments using fastIBD. Following the authors’ recommendations, IBD detection was conducted 10 independent times and then combined, retaining only segments supported by at least two independent runs and at least one segment reaching a confidence score below 10^−10^. We dropped approximately 1.3% of the segments that would otherwise be merged by criteria adopted by others who have similarly analyzed IBD segments detected by fastIBD (B. L. Browning and S. R. Browning 2013b; Ralph and Coop 2013; Durand *et al.* 2014), as we found that much of the need for merging may have come from genotyping errors or missingness, which are not allowed in simulations here. A total of 558 IBD segments greater than 1 cM from one simulated region were detected.

Our initial run with IBDLD, using all 1000 diploid individuals for one simulated region, did not complete within 72 hr. Therefore, we randomly selected a subset of 500 diploid individuals for our evaluation. We used the method GIBDLD (–method GIBDLD) and chose to output only segments with length > 1000 kb (–length 1000); otherwise, default settings were used. A total of 643 IBD segments were detected.

We also tested PLINK [v1.07, (Purcell *et al.* 2007)]. As recommended, we first thinned the dataset so that no pair of SNPs within a window of 100 markers (moving at windows of 25 markers) would have an r^2^ > 0.2. However, estimation of IBD segments on the thinned dataset, using default settings, detected no segments. Less stringent thinning, increased marker density, a lowered minimum number of SNPs, or minimum segment length did not significantly improve detection of IBD segments using PLINK (data not shown). This is consistent with the lower power to detect IBD segments by PLINK as observed by others (S. R. Browning and B. L. Browning 2010), and PLINK was thus not further evaluated.

### Calling ground-truth IBD_ARG_ segments from simulated data

We next used the simulated ARGs to determine the true IBD segments. MaCS reports the ARG as a sequence of genealogical trees along the simulated region, so that neighboring trees differ if a recombination event has occurred somewhere in that genealogical tree. Such an event may not affect the IBD status of a given pair of diploids, if it does not involve any of their four haplotypes. An IBD_ARG_ segment shared by a pair of diploids is then defined as a continuous stretch of genealogies with unchanging age to common ancestor (*i.e.*, no recombination since the common ancestor) between at least one pair of the four pairwise haplotype configurations. This may conflate segments on which there has been an ancestral recombination but both common ancestors lived at the same time; but the chance of this is very small. We then aimed to identify all IBD_ARG_ segments between all pairwise comparisons of diploid individuals in the simulation. However, it is computationally challenging to enumerate all such segments in an ARG, so we implemented two approximations to make the problem computationally tractable. First, we sampled a genealogy only every 0.01 cM (or 10 kb) of the simulated dataset, greatly reducing the number of genealogies that need to be processed. Second, we extract only IBD_ARG_ segments less than 3000 generations old, greatly reducing the number of IBD segments to follow and keep in memory. Together, these approximations allowed us to obtain nearly all segments with length ≥ 0.2 cM at a cost of uncertain boundary resolution within 0.01 cM, as none of > 5.7 million segments have age very close to 3000 generations (Supplemental Material, Table S1). While our approach may not detect all IBD_ARG_ segments in the simulated dataset (especially the very short ones), we can ensure that all detected IBD_ARG_ segments in this manner will be true IBD segments, free of any conflation effects.

These simulations produced rates of IBD somewhat lower than published, empirical estimates. Our main simulation adopts a European demography (Nelson *et al.* 2012) that assumed a panmictic population experiencing an explosive recent expansion reaching an effective population size of 4,000,000. We detected 4208 IBD_ARG_ segments > 1cM among 1000 simulated individuals, which translates to an IBD rate of 8.42 × 10^−3^ segments per pair of individuals per 100 Mb region. For comparison, Ralph and Coop (2013) used fastIBD and detected ∼1,900,000 (IBD_called_) segments > 1 cM genome-wide in a population of ∼2200 general Europeans. This translates to an IBD rate of approximately 29.95 × 10^−3^ segments per pair of individuals. The difference in rates is likely due to unmodeled local population structure, which increases recent coalescence rates relative to a randomly mating population, as well as due to other differences such as imperfectly modeled demography, marker density, genotyping errors, and false positives of the calling algorithm.

### Estimating mutation rate based on IBD segments

There has been recent interest in estimating mutation rates based on principles of IBD sharing to complement existing approaches from trios and phylogenetic analyses (Campbell *et al.* 2012; Palamara *et al.* 2015). Here, we adopted a simplistic estimator of mutation rate based on IBD, similar in spirit to that used in Campbell *et al.* (2012) with autozygous segments, assuming that the phase and demographic history of the sample are known and that no genotyping or sequencing errors exist. We will show that without taking the conflation phenomenon into consideration, our mutation rate estimates will be biased. However, we note that the studies referenced here have utilized external information, such as pedigrees and trio-based phasing, to implicitly overcome the conflation problem; see *Discussion*.

Differences between IBD segments must have occurred more recently than the common ancestor giving rise to the IBD. Therefore, an estimator of the mutation rate μ is:

$$\mu =\;\frac{\sum_{i\in I}m(i)}{\sum_{i\in I}2L_{seq}(i)T_{IBD}(i)}$$ (1)

where *I* is the set of all IBD segments, and for a given IBD segment *i*, *m(i)* is the number of sequence mismatches on the two IBD haplotypes, *L_seq_(i)* is the total sequenced region (= the length of the IBD segment, *L_IBD_(i)* (if completely sequenced), and *T_IBD_(i)* is time since the common ancestor in generation.

### Simulating conflated segments to test the impact of conflation on mutation rate estimator

Based on all of the IBD_ARG_ segments with length > 0.2 cM, we first estimate the distribution of IBD segment ages given segment length in the simulation. As suggested by visual inspection, we binned lengths to 0.1 cM resolution, and for each length bin, fitted a mixture of two γ distributions to the true distribution of ages in that bin using an EM algorithm implemented in the R package mixtools (Table S2). In practice, this distribution will change depending on the underlying demography of the population and will likely be difficult to estimate. However, for the purpose of our illustration here, we assumed that the demography is known.

Then, to simulate conflated segments in a more efficient manner than using the coalescent, we sampled IBD segment lengths from the apparent length distribution and assigned each sampled IBD segment as a conflated segment or a nonconflated segment, depending on its length and the appropriate proportion of conflation given its length observed in the simulations. For nonconflated segments, we sampled an age, *T_UC_*, from the age distribution given its length, *L_UC_*, using the mixture γ (parameters given in Table S2). The number of sequence mismatches in each IBD segment were then sampled from a Poisson distribution with mean *2L_UC_T_UC_μ*, where μ is the mutation rate and set to 1.2 × 10^−8^.

For conflated segments, we make the simplifying assumption that the entire segment of length *L_C_* is composed of only two subsegments of length *L_C1_* and *L_C2_*, with no gap. *L_c_* is sampled from the apparent length distribution (with conflation), *L_C1_* is sampled from the true length distribution of IBD_ARG_ segments, and *L_C2_ = L_C_ – L_C1_*. For each pair of subsegments, we then sampled ages *T_C1_* and *T_C2_*, and sampled numbers of sequence mismatches as above.

For both conflated and nonconflated segments, we also sampled the apparent age based on the apparent length (the full length for nonconflated segments, and the sum of the two lengths for conflated segments). These will be the estimated *T_IBD_* used in Equation 1. We generated 1000 sets of 5000 IBD segments (roughly the number of segments > 1 cM detected by Refined IBD across the five 20 Mb regions), and report the median, fifth and 95th percentile estimates of the mutation rate.

Note that our strategy here is aimed at examining the potential impact of conflation, and conflation alone, on our mutation rate estimator in a conservative manner. As such, we assumed that there are no genotyping or sequencing errors, the phase and the demography are known, and that there is no misestimation of the IBD segment boundaries and no gaps between the conflated segments. In practice, these are all issues that can further confound the analysis.

### Data availability

Codes used for analysis in this paper are available upon request.

## Results

### The prevalence of subsegment conflations among called IBD segments

**Figure 2:**
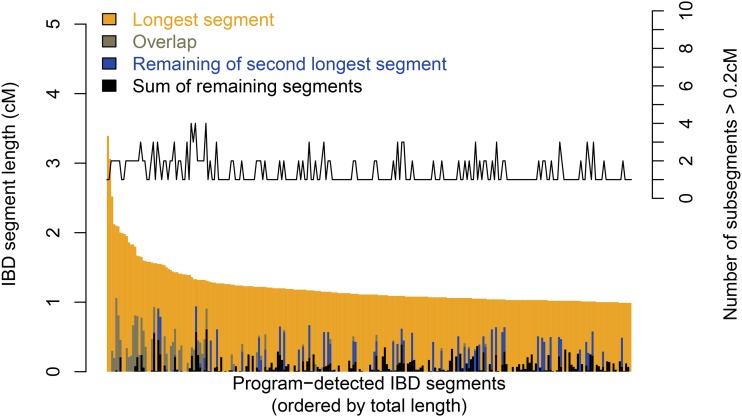
The prevalence of subsegments among algorithm-detected IBD segments. Each of a set of 250 randomly chosen IBD_called_ segments detected by Refined IBD is represented by a vertical bar. The IBD_called_ segments are sorted along the x-axis according to the detected length in decreasing order. For each IBD_called_ segment, the longest intersecting IBD_ARG_ segment is shown in yellow. The second longest intersecting IBD_ARG_ subsegment, if present, is shown in blue. The overlap between the two longest IBD_ARG_ segments, if any, is shown in olive green. The remainder of the detected region is clumped in black. For each IBD_called_ segment displayed, we also show the number of subsegments > 0.2 cM detected in simulation using the vertical axis on the right. For completeness, we also display cases where the second IBD_ARG_ segment is completely overlapping the longest IBD_ARG_ segment, in which case it would not confound the calling algorithm. See Figure S3 for results based on IBD_called_ segments detected by GERMLINE, fastIBD, and IBDLD. ARG, ancestral recombination graph; IBD, identity-by-descent.

**Figure 3:**
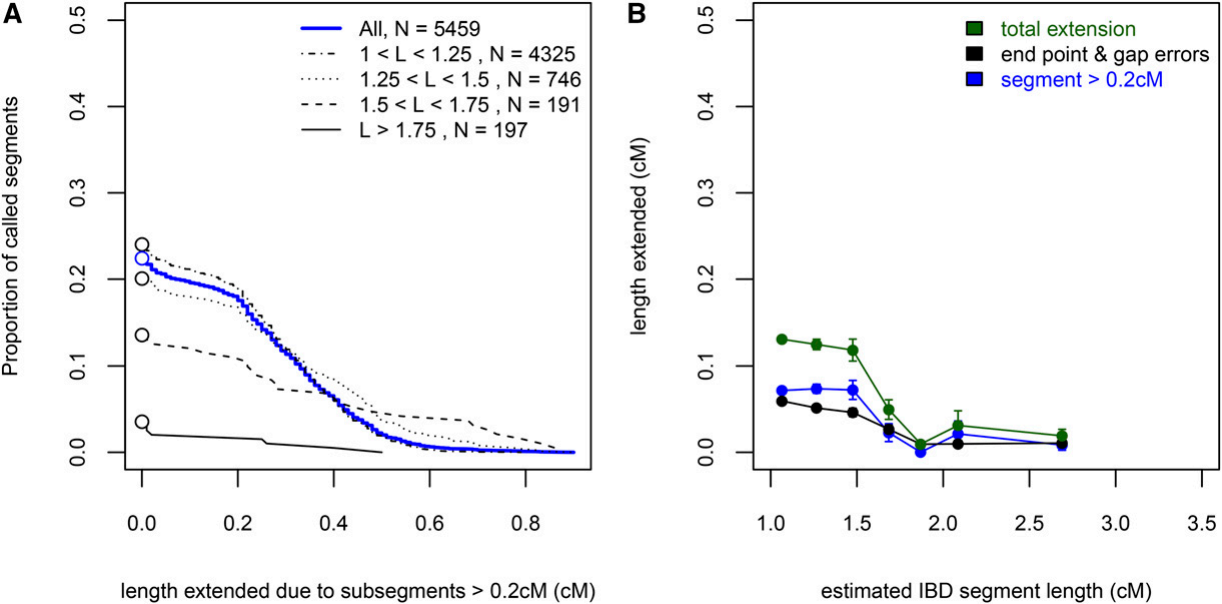
The conflation effect as a function of the length of IBD_called_ segments. (A) The complementary cumulative distribution functions (*i.e.*, 1-CDF) for the total length extended due to subsegments > 0.2 cM (except for the longest IBD_ARG_ segment). The distributions are also stratified by four levels of length: Between 1–1.25 cM, between 1.25–1.5 cM, between 1.5–1.75 cM, and > 1.75 cM. The conflation effect is generally driven by segments < 1.75 cM in detected length. (B) The biases in estimated length due to subsegments and end point errors as a function of the estimated length. We binned all IBD_called_ segments in 7 bins: [1, 1.2), [1.2, 1.4), [1.4, 1.6), [1.6, 1.8), [1.8, 2), [2, 2.2), [2.2, 20), and for each bin examined the average length extended (from both ends) beyond the longest IBD_ARG_ segment found in the called region due to either a subsegment > 0.2 cM (blue), or other minor endpoint errors and gaps between subsegments (black). Each data point is plotted on the x-axis at the median length of the bin. ARG, ancestral recombination graph; CDF, cumulative distribution function; IBD, identity-by-descent.

**Figure 4:**
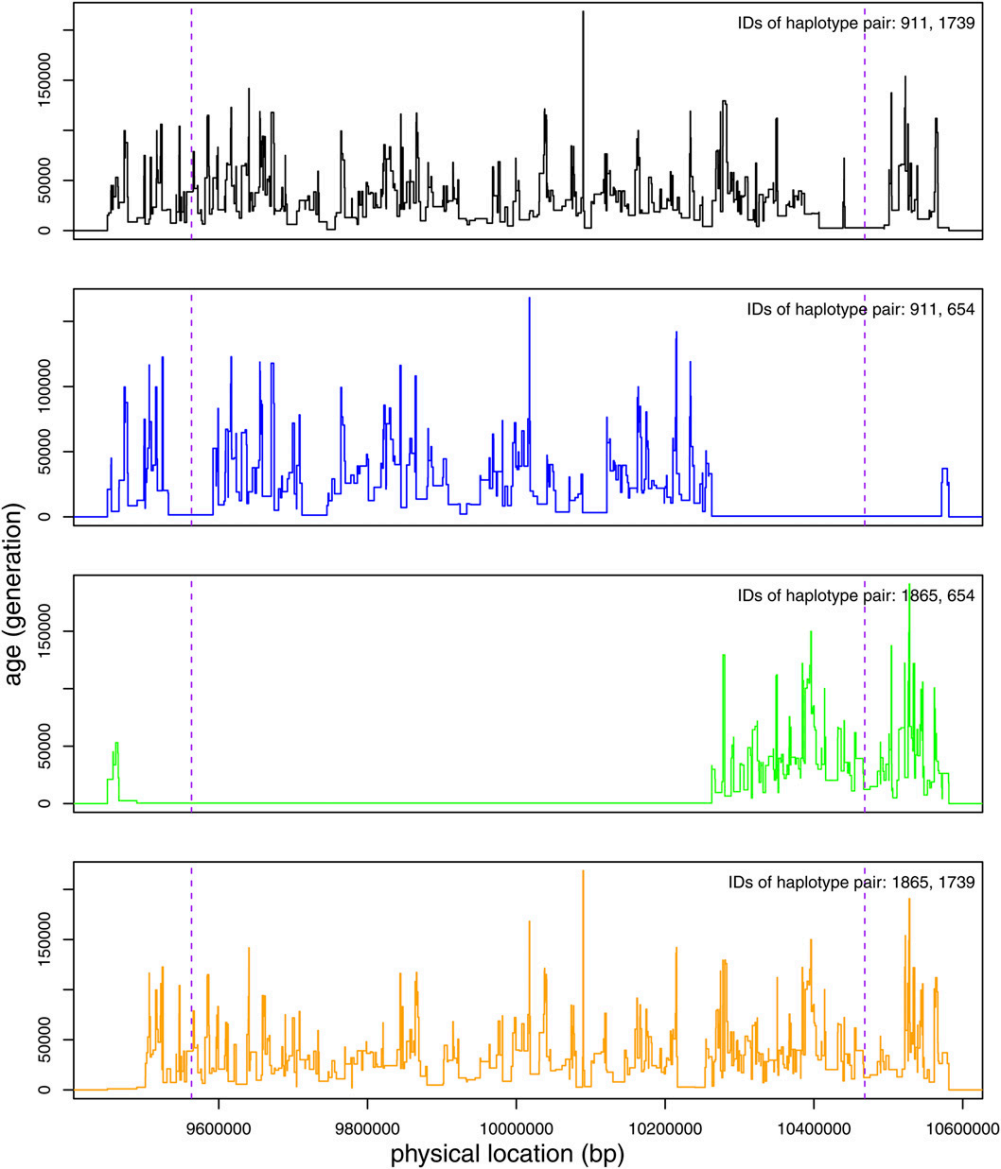
An illustrative example of the conflation effect on the mutation rate estimator. For a particular IBD_called_ segment of length 1.145 cM, we show the distribution of IBD_ARG_ subsegment age (y-axis) as a function of position (x-axis, between 9.45–10.59 Mb of a 20 Mb simulated region). Each of the four different pairwise haplotype configurations between the two diploid samples is illustrated with a different color. The simulated haplotype numbers are displayed in the upper right hand corner. The vertical dashed lines demarcate the 10% segment length from both ends of the segment that one could remove from analysis due to the uncertainty in estimating the ends of the IBD_called_ segments. The age of each subsegment is plotted as a step function of its length. In this case, the IBD region is dominated by two long segments of IBD, one between simulated haplotypes 1865 and 654, another between simulated haplotypes 911 and 654. (There is actually a third, very short, segment of recent coalescence between simulated haplotypes 911 and 654 that is not obvious here.) Regions that do not produce long IBD segments can be clearly seen with the deep coalescences. In this case, the predominate IBD haplotype should be between haplotypes 1865 and 654, but the conflation with a neighboring IBD haplotype between haplotypes 911 and 654 led to the estimation of a single long IBD segment. ARG, ancestral recombination graph; IBD, identity-by-descent.

Across the simulations, Refined IBD called a total of 5459 IBD_called_ segments. A random subset of 250 IBD_called_ segments, together with how they are composed of IBD_ARG_ segments, is displayed in Figure 2; the entire dataset is displayed in Figure S1. Some of the subsegments completely overlapped with the longest IBD_called_ segment due to diploidy, but 22.4% of the IBD_called_ segments with length > 1 cM include portions of IBD_ARG_ segments that are shorter but at least 0.2 cM long, 17.5% of the IBD_called_ segments have their length extended by at least 0.2 cM (Figure 3A). When extended, the length of the IBD segments is extended by an average of 0.31 cM. One illustration of a conflated segment is shown in Figure 4, where between the four possible pairs of haplotypes between two diploid individuals, two separate segments were inherited from two sets of recent, but different, common ancestors that are in close proximity to each other, leading the algorithm to call the entire region IBD.

Comparing segment length biases due to conflations as a function of the called segment length, we see that the contribution due to conflation of subsegments > 0.2 cM is greater than that due to minor endpoint errors and gaps in the calling (defined here as the regions of the called segment that are not attributed to a subsegment > 0.2 cM) (Figure 3B). The minor endpoint and gap errors are relatively independent of the length of the IBD_called_ segment length, while biases due to conflation are concentrated among the shorter segments (< 1.75 cM, Figure 3, A and B). Discarding 0.1 cM from either end of the IBD_called_ segments improved the calling slightly, reducing the proportion of conflated segments to 19.9% (12.9% of segments have their length extended by at least 0.2 cM), with an average of 0.26 cM increase in length when a segment is extended. However, the improvement is mostly due to reducing the minor endpoint errors, with little impact on the errors due to conflation (Figure S2). Together, these results suggest that a significant contributor to the biases in the length of IBD_called_ segments is the conflation of subsegments that are themselves stretches of IBD inherited from a relatively recent, though different, common ancestor. The conflation error also differs from the typical minor inaccuracies of detecting endpoints, in that the overlap with a true segment does not improve if we discard the ends of the called segment.

While we have thus far focused on IBD_called_ segments produced by the Refined IBD program, our observation should not be specific to Beagle, but is more generally applicable to any IBD calling algorithm so long as these programs depend solely on sequence identity for inference. To test this hypothesis, we also used fastIBD (B. L. Browning and S. R. Browning 2011), GERMLINE (Gusev *et al.* 2009), and IBDLD (Han and Abney 2013) to call IBD segments in each of our simulated regions. We observed the same conflation effects across these algorithms, though different algorithms were affected to different degrees (Figure S3 and Figure S4).

Finally, the conflation effect is also not specific to the demography we simulated. In addition to the European-like human demography estimated in Nelson *et al.* (2012), we also simulated under both the European and African demographic histories published in Tennessen *et al.* (2012), an alternative European-like model without the most recent exponential growth phase, as well as a constant size population. Though different demographic scenarios are affected by the conflation events to different degrees, the conflation effect is evident in all cases (Figure S5).

### The rate of conflation of true IBD segments in the simulated ancestral recombination graphs

Though we have observed significant conflation among IBD segments called by a number of algorithms, it remains possible that this is particular to the technical aspects of the calling algorithm, and avoidable somehow. Thus, we next characterized the rate of conflation events between true IBD segments identified from the simulated ancestral recombination graph. We define a conflation event in our simulated data as when two IBD_ARG_ segments, each of length at least *w* cM, separated by a gap no greater than a small number 0 (two subsegments overlap) or 0.01 cM, together account for an end-to-end length of at least 1 cM. The simplest model of IBD segment conflation is that, given the length distribution determined by the coalescent time distribution, observed long IBD segments are uniformly and independently distributed along the genome. To test this, we simulated 100 replicate datasets by permutation where we assigned each segment randomly (with replacement) to two diploid individuals uniformly across the genomic regions.

**Figure 5:**
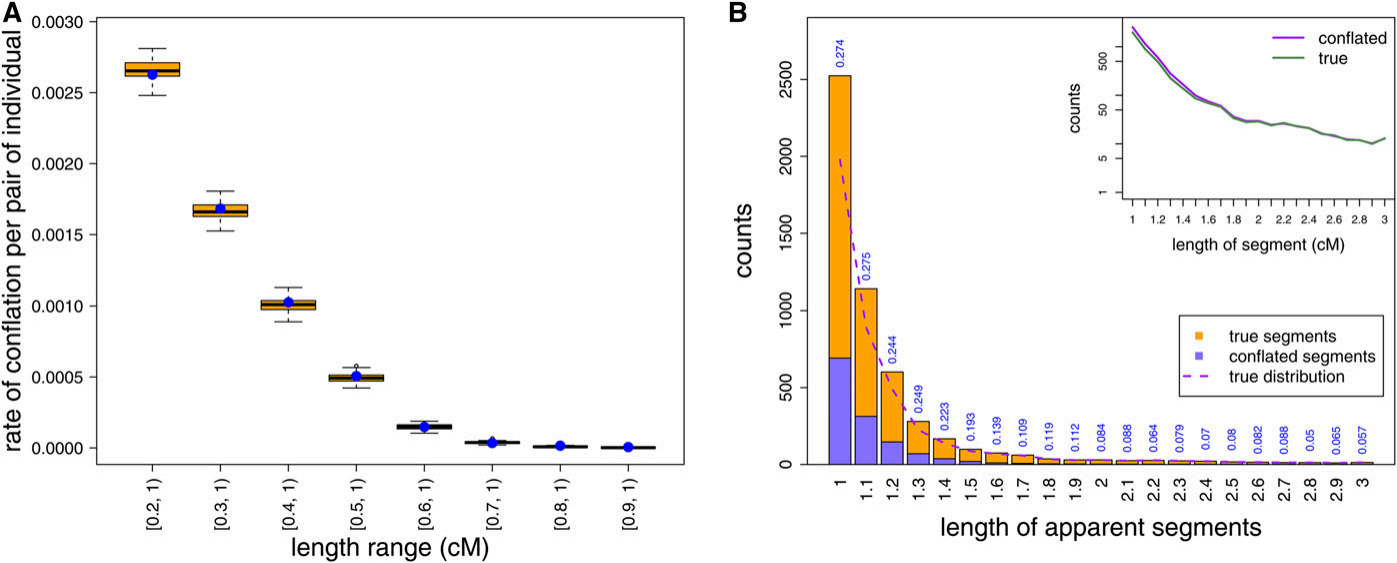
Conflations of shorter IBD segments will bias the length distribution. (A) For each bin of segment length range, we calculated the rate at which two IBD_ARG_ segments in our simulations, both within the length range, are adjacent and together constitute an end-to-end length of at least 1 cM (*i.e.*, maximum gap sizes = 0 cM and combined length > 1 cM). The blue dot is the actual value observed in simulation. The boxplot shows the variance around the observed value by randomly sampling from the observed segment length distribution but randomly assigning the location of a segment and sample IDs 100 times. (B) The biased length distribution if each conflated IBD_ARG_ segment is counted for its conflated length rather than the two true lengths. Note that the apparent length of each conflated segment is due to conflation of two IBD_ARG_ segments, independent of any imprecision due to algorithm calling. Dotted line is the true length distribution if each conflated segment can be resolved based on the coalescent genealogy. Inset shows the comparison between the biased length distribution and the true length distribution in log scale. For results based on a maximum gap size of 0.01 cM, refer to Figure S6. ARG, ancestral recombination graph; IBD, identity-by-descent.

We observe that the conflation rate increases substantially as increasingly smaller IBD_ARG_ segments are allowed (Figure 5A and Figure S6). When counting conflation events due to segments as small as 0.2 cM, the rate of conflation (2.66 events per 1000 pairs of individuals per 100 Mb) is actually on the same order of magnitude as the true IBD rate > 1 cM in length (8.42 events per 1000 pairs of individuals per 100 Mb) in the simulated dataset. When compared with the simulated replicates, the levels of conflation are comparable (Figure 5A), suggesting that the majority of the observed conflations are due to the random independent placement of IBD segments along chromosomes. Therefore, any potential correlation in long segment placement induced by the haploid coalescent or the pedigree structure appears minimal.

To further investigate this, one can also assign each pair of conflated segments as being in *cis* or *trans* based on whether the pairs involved 2, 3, or 4 of the possible four haplotypes: a pair is *cis* if both IBD segments were shared by the same two (out of the four possible) haplotypes, implying that the segments descend from recent but different common ancestors; the pair is *trans* if the pairs involve three or four of the possible haplotypes (Figure S7). In theory, *trans* conflations should only be problematic to a haploid-based IBD detection algorithm if phasing is imperfect, and thus represent the amount of conflation problems that can be avoided with better phasing. We find that approximately 80% of the conflations occur *in trans* rather than *in cis* (2.13 events *vs.* 0.498 events per 1000 pairs of individuals per 100 Mb). As there are three times as many possible *trans* arrangements as *cis*, the proportion predicted by independent placement, 75%, is within the confidence interval.

The observed rate of conflation leads to a noticeable bias in the length distribution of apparent IBD segments with length greater than 1 cM (Figure 5B and Figure S6). Specifically, approximately 27% of the apparent IBD segments between 1–1.2 cM long are due to conflation.

### Evaluating the impact of conflation on a mutation rate estimator

We evaluated the impact of the biased length distribution of IBD segments due to conflation on a mutation rate estimator. Given accurately defined IBD segments and perfect sequence and phase information, the sequence mismatches between a pair of individuals in the IBD segment reveals mutations that have arisen since the common ancestry of the two IBD haplotypes. Therefore, we devised a simplistic mutation rate estimator based on observed sequence mismatches between a pair of IBD haplotypes and a conservative heuristic to generate simulated data (*Methods*).

When we applied the mutation rate estimator on a set of nonconflated segments > 1 cM (*i.e.*, all IBD segments are called perfectly), we would estimate the same mutation rate (1.201 × 10^−8^/bp/gen; fifth and 95th percentiles = 1.174 × 10^−8^ and 1.228 × 10^−8^ /bp/gen, respectively) as the value used to simulate the data (1.20 × 10^−8^ /bp/gen). When the estimator is applied on a set of conflated segments, all composed of two subsegments, we would estimate μ to be 1.647 × 10^−8^ /bp/gen (fifth and 95th percentiles = 1.596 × 10^−8^ and 1.702 × 10^−8^ /bp/gen, respectively), which is a 37% inflation. When we applied the estimator to a mixture set composed of ∼34% conflated segments (as would be predicted by Figure 5B), the estimated μ is 1.311 × 10^−8^ /bp/gen (fifth and 95th percentiles = 1.287 × 10^−8^ and 1.337 × 10^−8^/bp/gen), a more modest 9% inflation. As one increases the length cut-off for IBD segments for analysis, the median estimate of μ becomes less inflated, though still higher than the true value due to the persistent low level of conflation, and with a wider confidence interval due to fewer IBD segments for analysis (Table S3).

## Discussion

A number of population genetic analyses focus on the information provided by the IBD segment length distribution. So far, nearly all applications assume that each detected IBD segment is derived from a single common ancestor. However, the observed IBD length distribution is often biased, and extensive efforts are undertaken to empirically correct the observed distribution in order to ensure the validity of the downstream conclusions. In this manuscript, we studied this core assumption, and examined through simulations the phenomenon of conflation of shorter segments. Our characterization of this phenomenon shed light on one of the mechanisms by which the IBD length distribution is biased in practice.

We have shown that using simulated SNP array data, under a reasonable demography for western European humans, and irrespective of the choice of IBD-calling algorithm examined, an appreciable proportion of called IBD segments shorter than 2 cM are composed of conflations of shorter, more distantly related, subsegments. For instance, of segments > 1 cM inferred using Refined IBD, greater than 22% have the IBD segment length extended due to other short subsegments > 0.2 cM; most of these were extended by 0.2–0.5 cM. The rate of conflation is reduced to ∼6% for longer IBD segments (> 2 cM) (Figure 3A). In our simulations, chance conflations often arose between independent, shorter segments (*e.g.*, two of sizes 0.5 cM); this creates more segments near the shorter end of our detection threshold (1 cM) than longer because the true segment length distribution is so strongly peaked near zero (Figure 5). Furthermore, we have shown that the conflation errors are unlike typical minor endpoint estimation errors, which would be improved by removing ends of the segments from downstream analysis (Figure 3B and Figure S2). However, we note that the severity of the conflation problem may differ in real data, as other factors such as the marker density and the underlying, unobserved population demography could also be in play.

The conflation effect described here could potentially in part explain the recent observation of pervasive false-positive IBD segments detected in pedigrees (Durand *et al.* 2014). In Durand *et al.* (2014), the authors noted that over 67% of the IBD segments they identified with GERMLINE between a child in a trio to an unrelated individual are not observed between either of the parents in the trio to the same unrelated individual. This false positive effect is also exacerbated near the algorithm’s length threshold for making a call. Conceivably, at least a subset of these false positive events is not due to blatant errors in the calling algorithm. Instead, some of the IBD segments called in the child-to-other comparisons could be due to conflations of IBD segments, independently inherited from the two parents. As a result, the full-length IBD segments are not observed in either of the parents. However, also note that implementation differences of the pipelines utilizing GERMLINE could cloud direct comparisons. For example, Durand *et al.* (2014) used different length thresholds as used here, and did not use the haploid mode of GERMLINE, which is expected to improve performance.

The failure to account for the biased length distribution of IBD segments due to conflation would lead to errors in downstream analysis, which we demonstrated with a simplistic estimator of the mutation rate based on IBD segments. While we focus only on the issue of conflation, other hurdles exist to estimating mutation rates from IBD segments, such as estimating the age of the MRCA or dealing with imperfect computational phasing. Indeed, utilizing external information (such as focusing on autozygous segments or using trio-phasing) to overcome these issues, as well as modeling of genotyping errors, are the focus of recent attempts to estimate mutation rate using the IBD concept (Campbell *et al.* 2012; Palamara *et al.* 2015). However, the external information used by these studies is not always available, and would reduce the sample sizes available for analysis. As such, there remains room for improvement.

Other types of analyses that rely heavily on the length and age distribution of IBD segments are demographic inferences based on IBD segments. For example, Ralph and Coop (2013) devised a nonparametric approach to infer the number and age of recent shared ancestors between populations based on IBD sharing between populations. This provides estimates of distributions of the ages of IBD segments given their lengths, but analytic difficulties inherent to demographic inference may make these quite noisy. Palamara *et al.* (2012) avoided this by fitting demographic models parameterized with few parameters. In both cases, it would be preferable to directly model conflation of short segments, although both carefully corrected the observed length distribution of IBD segments to reach robust conclusions. Ralph and Coop (2013) focused only on IBD segments longer than 2 cM, which we show could remove the majority of conflation effects, and further modeled the power, false-positive rates, and length misestimation of IBD detection. Instead of using a hard cut-off, Palamara *et al.* (2012) used an approach that iterates between demographic inference and IBD detection, until they converged on a set of parameters for GERMLINE that minimized the difference between called and true IBDs from simulation specific to the data at hand, prior to the formal demographic inference procedures. Alternative approaches using IBS directly rather than IBD can also be taken, as the length of IBS segments can be more precisely obtained given high-coverage sequencing data (Harris and Nielsen 2013). It will be interesting to investigate if a theoretical framework contrasting IBS and apparent IBD segments can predict the rate of conflated segments within a dataset.

Though we focused on an IBD detection framework using high-density array data, as the field develops algorithms aimed to detect even shorter segments due to the improved density of markers in the era of whole genome sequencing [*e.g.*, programs like IBDseq (B. L. Browning and S. R. Browning 2013a)], it seems imperative to note and reevaluate the potential problem of conflation. We found that ∼75–80% of the conflated segments are the result of pairs of segments existing *in trans*, suggesting that improvements in phasing may greatly reduce, but not completely eradicate, biases due to conflated segments. Therefore, in addition to improvements in phasing, considerations of the conflation effect and external information such as availability of other individuals in the pedigree could all contribute toward improved accuracy in IBD detection and interpretation.

## Supplementary Material

Supplemental Material

## Appendix

### MaCS Commands for Simulations

The primary demographic model used throughout the manuscript is one based on deep resequencing of 202 genes across 14,000 European individuals (Nelson *et al.* 2012): the population has ancestral size of 12,500 up until 17,000 generations ago, 24,500 between 3500–17,000 generations ago, 7700 between 368–3500 generations ago, and exponentially expanding at a rate of 0.017 per generation to a present day size of 4 × 10^6^. The MaCS command used is:

macs 2000 20000000 -T -t 0.192 -r 0.16 -G 0.017 -eN 0.000023 0.001925 -eN 0.00021875 0.006 -eN 0.0010625 0.003125

All input parameters are scaled by 4N_0_ for MaCS, with N_0_, the present day effective population size, equaling 4 × 10^6^ in this case.

Moreover, we simulated under a number of other demographic scenarios, primarily based on those published by Tennessen *et al.* (2012). These include: the European and African demography from Tennessen *et al.* (2012), a European model but without the aggressive second exponential growth phase, as well as a constant size population. Specifically, the African demography was modeled with a population of size 7310 until 1480 generations ago, 14,474 between 205–1480 generations ago, and then exponentially growing at rate 0.0165 per generation until reaching a present day size of 424,000. For the European demography, we modeled a population with ancestral size 7310, that has a population size of 14,474 between 2040–5920 generations ago, 1861 between 920–5920 generations ago, after which the population experienced exponential growth at rate 0.003 per generation until 204 generations ago, when the growth rate increases to 0.0195 per generation to reach a present day size of 496,500. The alternative European model without the aggressive second exponential growth phase is similar, but reaching only a present day size of 9300. Finally, the constant size population has a constant size of 100,000. The MaCS command for these simulations are:

Tennessen European model (N_0_ = 496,500):

macs 2000 20000000 -T -t 0.023832 -r 0.01986 -G 0.0195 -eG 0.000102718 0.00307 -eN 0.000463746 0.00374823 -eN 0.00102719 0.02915204 -eN 0.002980864 0.01472305

Tennessen African model (N_0_ = 424,000):

macs 2000 20000000 -T -t 0.020352 -r 0.01696 -eG 0.0 0.016475022 -eN 0.000120873 0.034136873 -eN 0.003490568 0.017240607

Tennessen European alternate model (N_0_ = 9300):

macs 2000 20000000 -T -t 0.0004464 -r 0.000372 -eN 0.0 1.0 -eG 0.005510753 0.003074847 -eN 0.024758065 0.20010753 -eN 0.05483871 1.55634409 -eN 0.159139785 0.78602151

Constant size population model (N_0_ = 100,000):

macs 2000 20000000 -T -t 0.0048 -r 0.004 -eN 0.0 1.0
