# Supplementary material for: Conflation of Short Identity-by-Descent Segments Bias Their Inferred Length Distribution
Source: G3 (Bethesda). 2016 Mar 1;6(5):1287–96. doi: 10.1534/g3.116.027581 (PMC4856080; doi:10.1534/g3.116.027581)
Supplement: Supplemental Material [file supp_6_5_1287__index.html]

Conflation of Short Identity-by-Descent Segments Bias Their Inferred Length Distribution — Supplemental Material 

# Conflation of Short Identity-by-Descent Segments Bias Their Inferred Length Distribution

## Supplemental Material for Chiang, Ralph, and Novembre, 2016

**Files in this Data Supplement:**

- Figure S1 - The prevalence of subsegments among IBDcalled segments by Refined IBD. (.pdf, 374 KB)
- Figure S2 - The conflation effect as a function of the length of IBDcalled segments, after trimming 0.1cM from both ends of the IBDcalled segments. (.pdf, 13 KB)
- Figure S3 - The prevalence of subsegments among IBDcalled segments detected by fastIBD, GERMLINE, and IBDLD.(.pdf, 351 KB)
- Figure S4 - Comparison of the conflation effect among IBD segments called by a number of other algorithms. (.pdf, 10 KB)
- Figure S5 - Comparison of the conflation effect among simulations of different demographic histories. (.pdf, 8 KB)
- Figure S6 - Conflations of shorter IBD segments will bias the length distribution. (.pdf, 222 KB)
- Figure S7 - Illustration of *cis* vs. *trans* conflations. (.pdf, 320 KB)
- Table S1 - Summary of segment age binned by length. (.pdf, 224 KB)
- Table S2 - Parameter estimates of the age distributions by binned length as a function of mixture of two gamma distribution. (.pdf, 231 KB)
- Table S3 - Mutation rate estimates using different length cut-offs. (.pdf, 215 KB)
