# Supplementary material for: Conflation of Short Identity-by-Descent Segments Bias Their Inferred Length Distribution
Source: G3 (Bethesda). 2016 Mar 1;6(5):1287–96. doi: 10.1534/g3.116.027581 (PMC4856080; doi:10.1534/g3.116.027581)
Supplement: Supplemental Material [file supp_g3.116.027581_FigureS1.pdf]

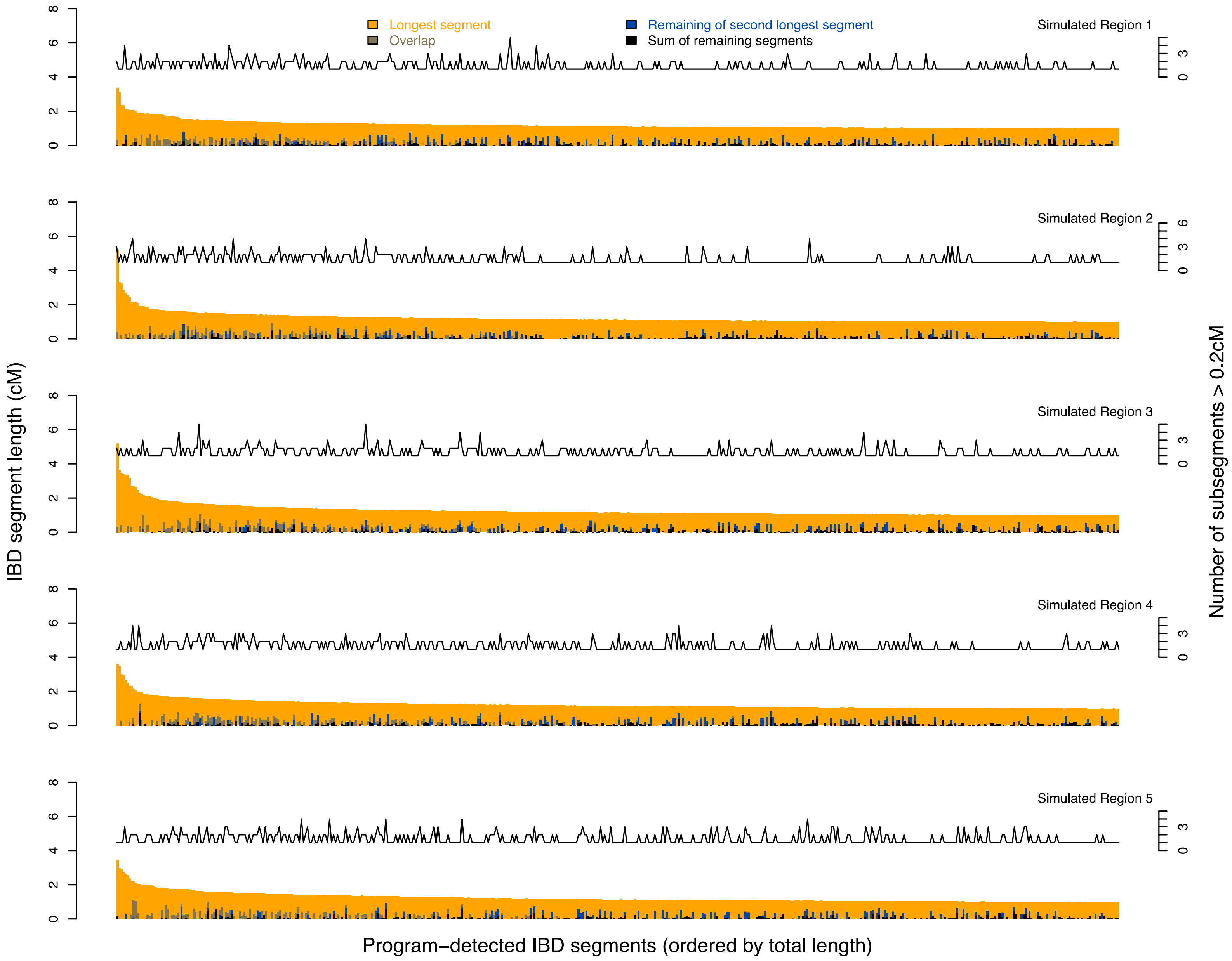
Longest segment
Overlap
Remaining of second longest segment
Sum of remaining segments
Simulated Region 1
Simulated Region 2
Simulated Region 3
Simulated Region 4
Simulated Region 5
IBD segment length (cM)
Number of subsegments > 0.2cM
Program−detected IBD segments (ordered by total length)

Figure S1: The prevalence of subsegments among IBD_{called} segments by Refined IBD

For ease of viewing, each barplot displays a random sample of 500 IBD segments detected by Beagle from each of the five simulated regions. Please refer to the legend of **Figure 2** for the color annotation of each IBD segment.
