# Supplementary figures and images for: Conflation of Short Identity-by-Descent Segments Bias Their Inferred Length Distribution
Source: G3 (Bethesda). 2016 Mar 1;6(5):1287–96. doi: 10.1534/g3.116.027581 (PMC4856080; doi:10.1534/g3.116.027581)

A

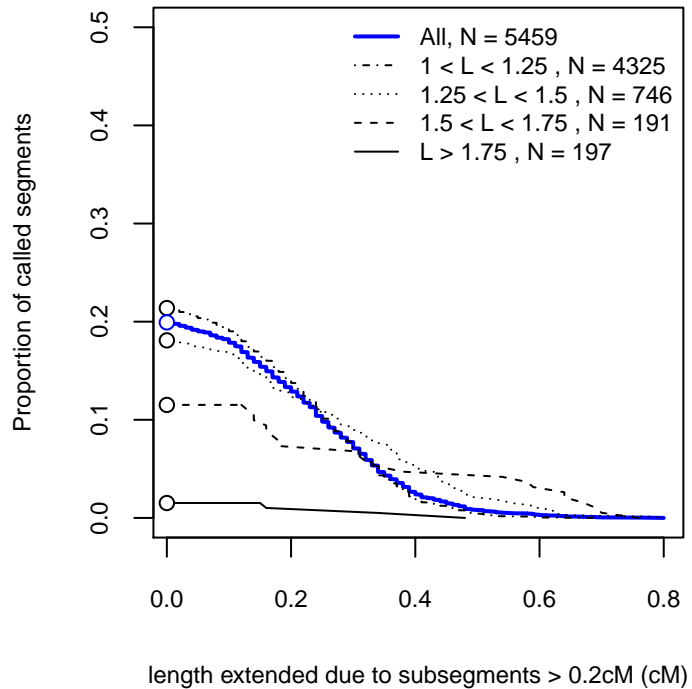

B

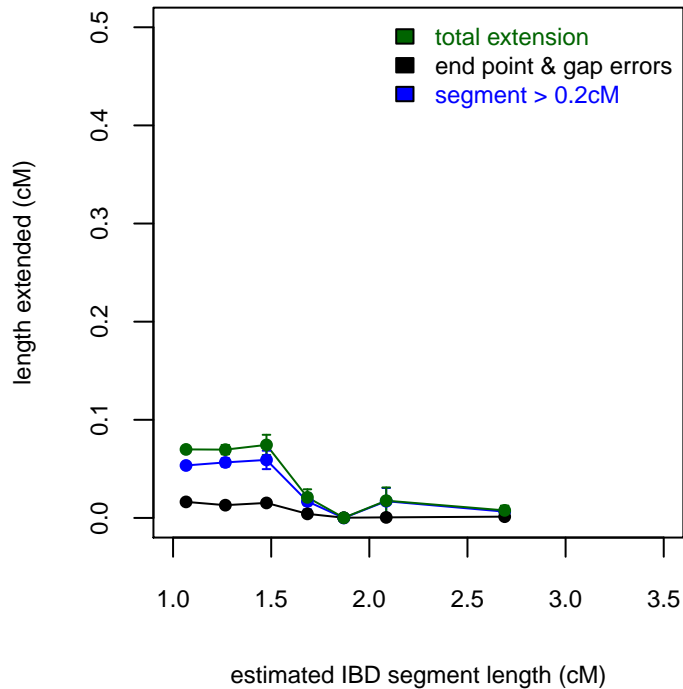

Supplement: Supplemental Material [file supp_g3.116.027581_FigureS2.pdf]

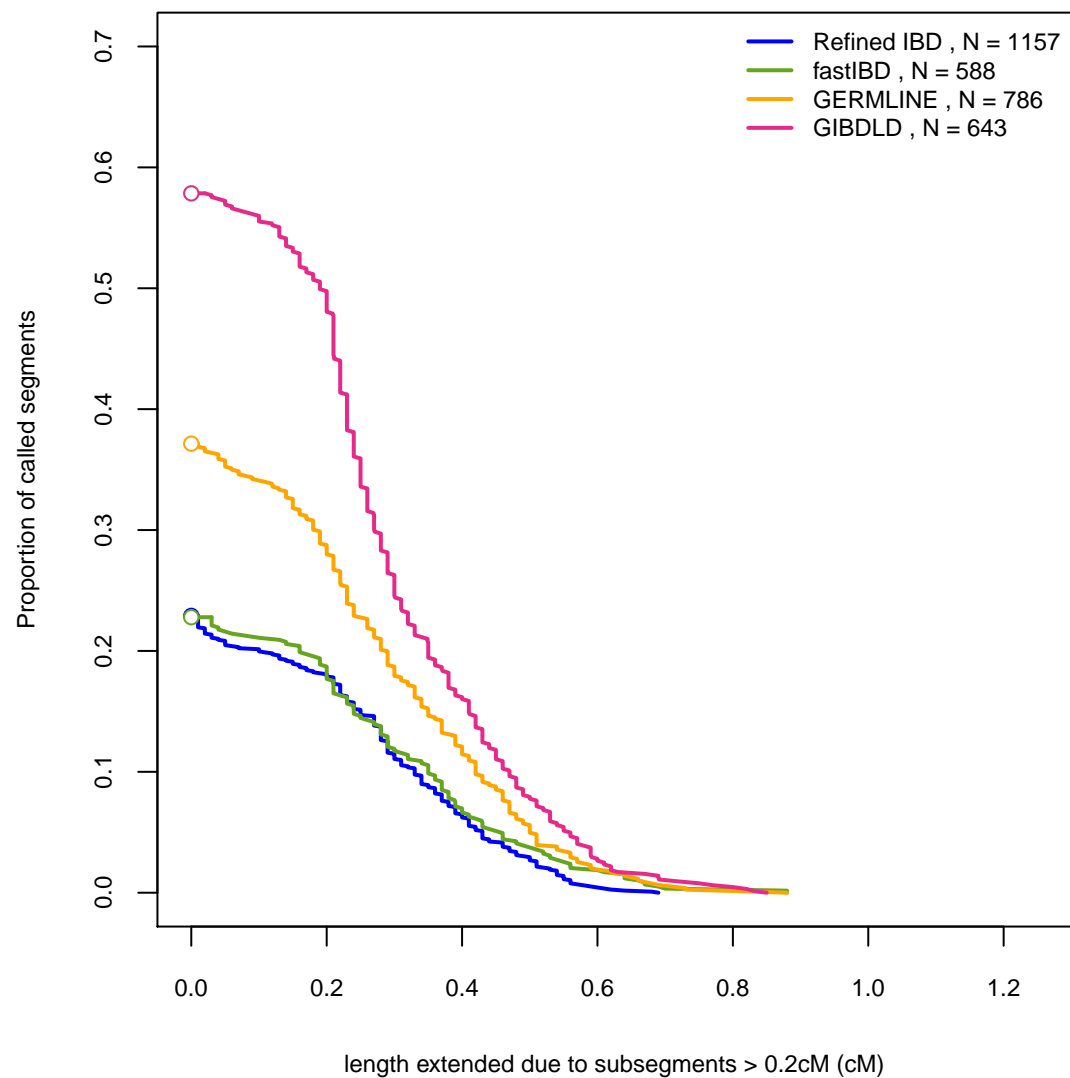

Supplement: Supplemental Material [file supp_g3.116.027581_FigureS4.pdf]

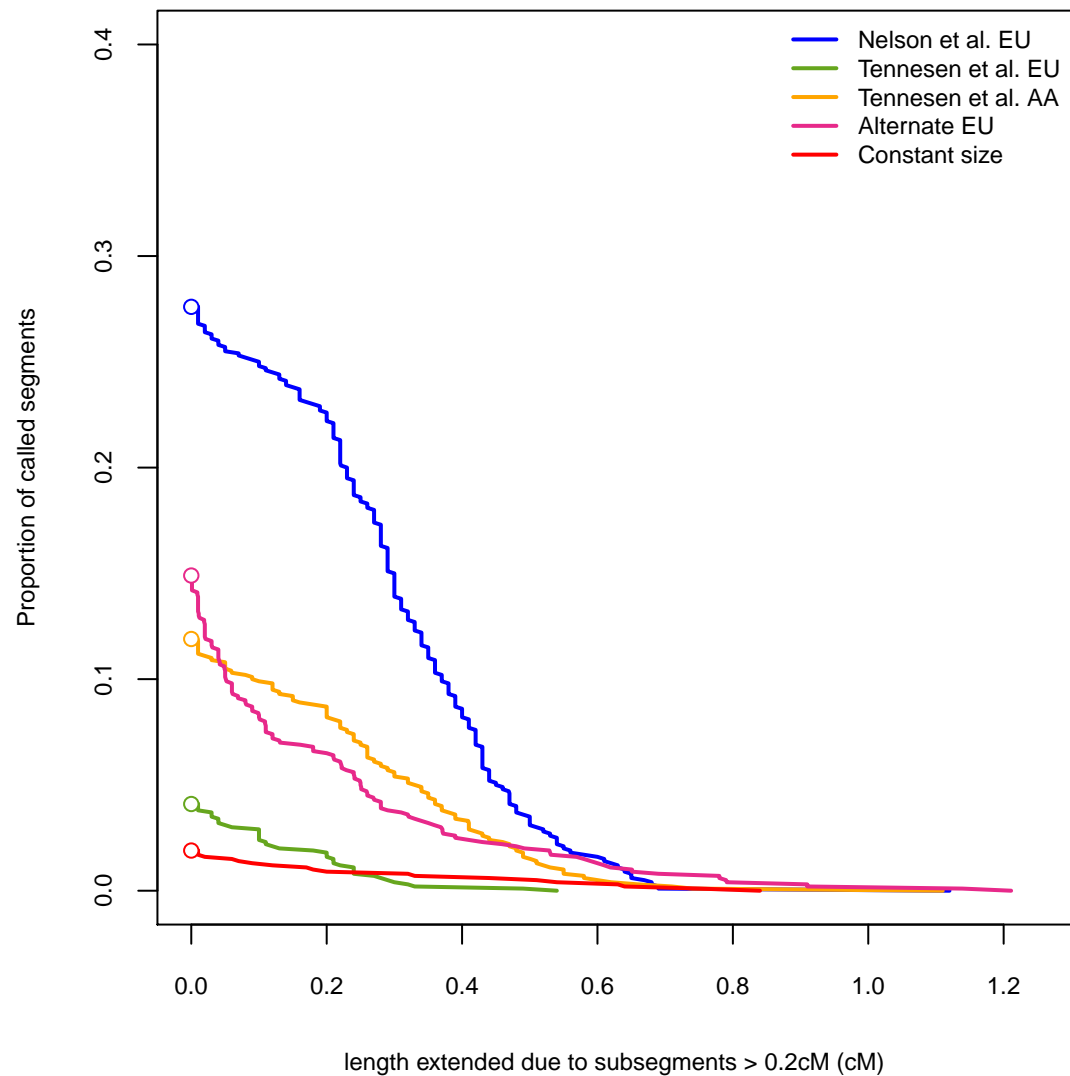

Supplement: Supplemental Material [file supp_g3.116.027581_FigureS5.pdf]
