# Supplementary material for: Conflation of Short Identity-by-Descent Segments Bias Their Inferred Length Distribution
Source: G3 (Bethesda). 2016 Mar 1;6(5):1287–96. doi: 10.1534/g3.116.027581 (PMC4856080; doi:10.1534/g3.116.027581)
Supplement: Supplemental Material [file supp_g3.116.027581_FigureS3.pdf]

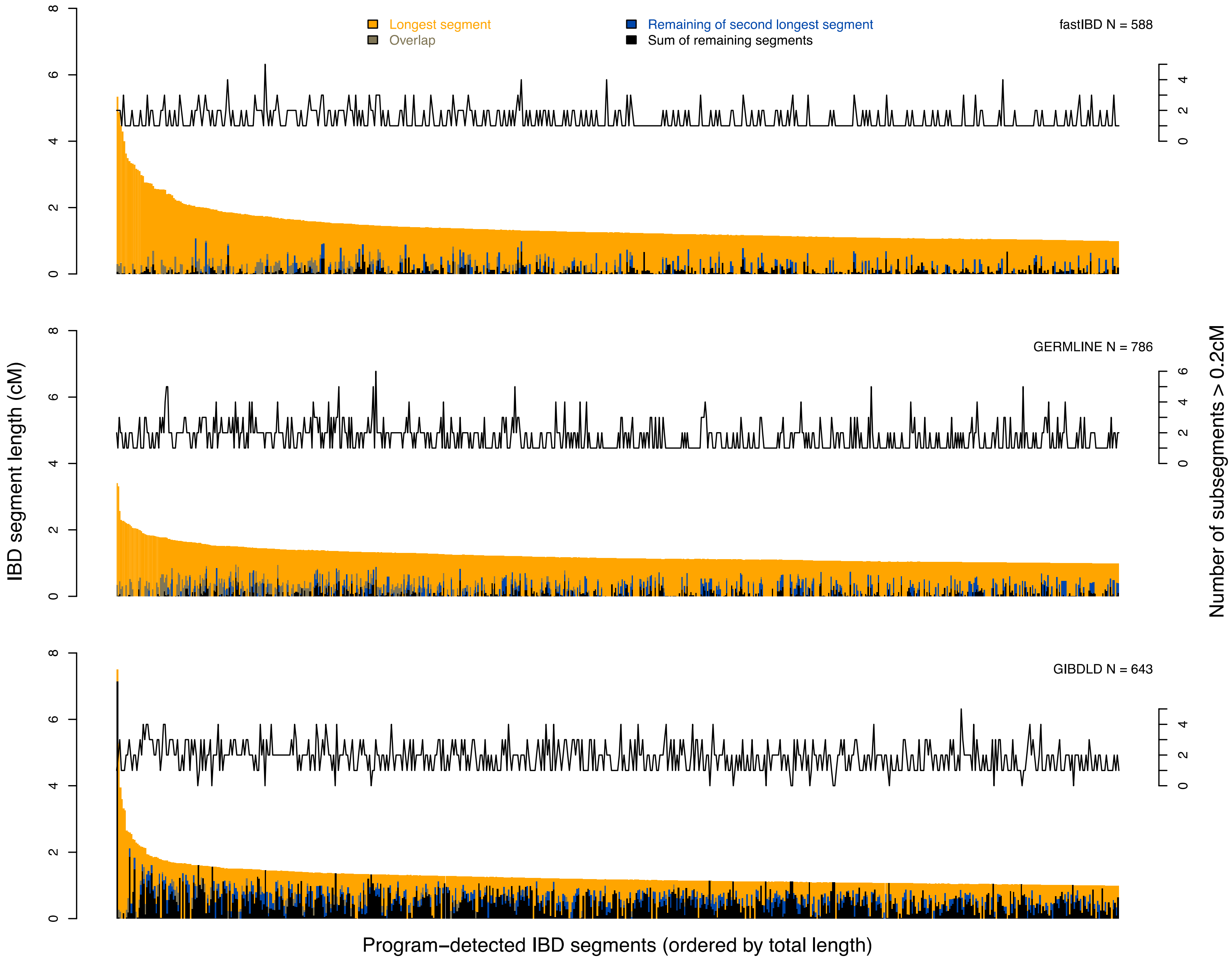
Longest segment
Overlap
Remaining of second longest segment
Sum of remaining segments
fastIBD N = 588
GERMLINE N = 786
GIBDLD N = 643
IBD segment length (cM)
Number of subsegments > 0.2cM
Program−detected IBD segments (ordered by total length)
0
2
4
6
8

Figure S3: The prevalence of subsegments among IBD_{called} segments detected by fastIBD, GERMLINE, and IBDLD.

The barplots display the IBD_{called} segments detected by fastIBD, GERMLINE, and IBDLD from one of the simulated regions. Please refer to the legend of **Figure 2** for the color annotation of each IBD segment. IBDLD appears to perform the worst in detecting non-conflated IBD segments. However, we note that IBDLD attempts to detect IBD segments using an HMM that assigns each position in the genome to one of 9 possible IBD states between the two diploid individual. The increased number of hidden states compared to other algorithms likely results in the increased noise of the called segments.
