# Supplementary material for: Conflation of Short Identity-by-Descent Segments Bias Their Inferred Length Distribution
Source: G3 (Bethesda). 2016 Mar 1;6(5):1287–96. doi: 10.1534/g3.116.027581 (PMC4856080; doi:10.1534/g3.116.027581)
Supplement: Supplemental Material [file supp_g3.116.027581_FigureS6.pdf]

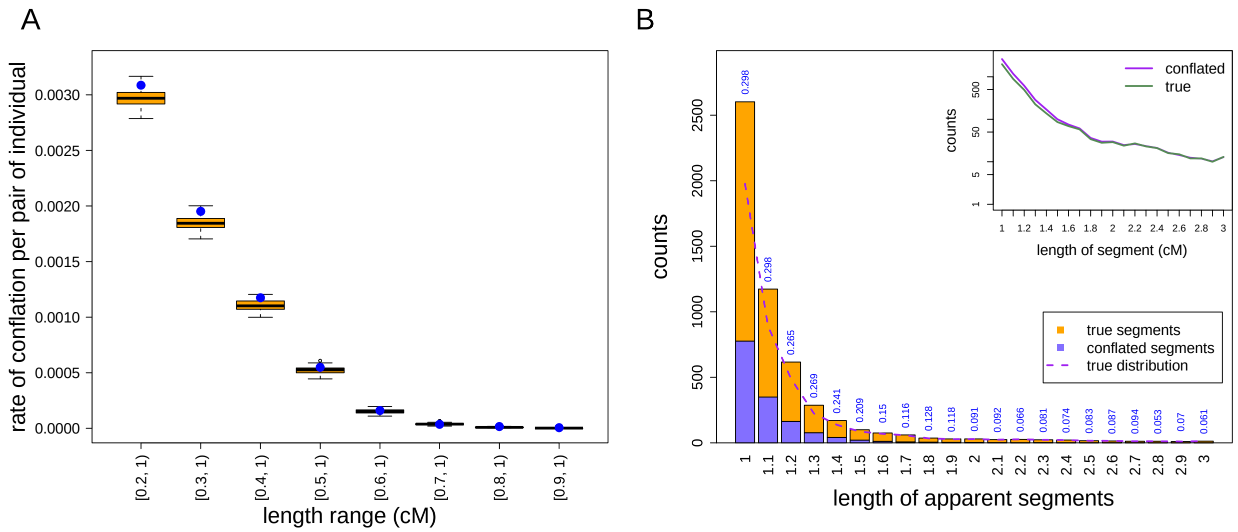

Figure S6: Conflations of shorter IBD segments will bias the length distribution

(A) For each bin of segment length range, we calculated the rate at which two IBD_{ARG} segments in our simulations, both within the length range, are separated no more than 0.01 cM and together constitute an end-to-end length of at least 1cM. The blue dot is the actual value observed in simulation. The boxplot shows the variance around the observed value by randomly sampling from the observed segment length distribution but randomly assigning the location of a segment and sample IDs 100 times. (B) The biased length distribution if each conflated IBD_{ARG} segment is counted for its conflated length rather than the two true lengths. Note that the apparent length of each conflated segment is due to conflation of two IBD_{ARG} segments, independent of any imprecision due to algorithm calling. Dotted line is the true length distribution if each conflated segment can be resolved based on the coalescent genealogy.
