# Supplementary material for: Conflation of Short Identity-by-Descent Segments Bias Their Inferred Length Distribution
Source: G3 (Bethesda). 2016 Mar 1;6(5):1287–96. doi: 10.1534/g3.116.027581 (PMC4856080; doi:10.1534/g3.116.027581)
Supplement: Supplemental Material [file supp_g3.116.027581_FigureS7.pdf]

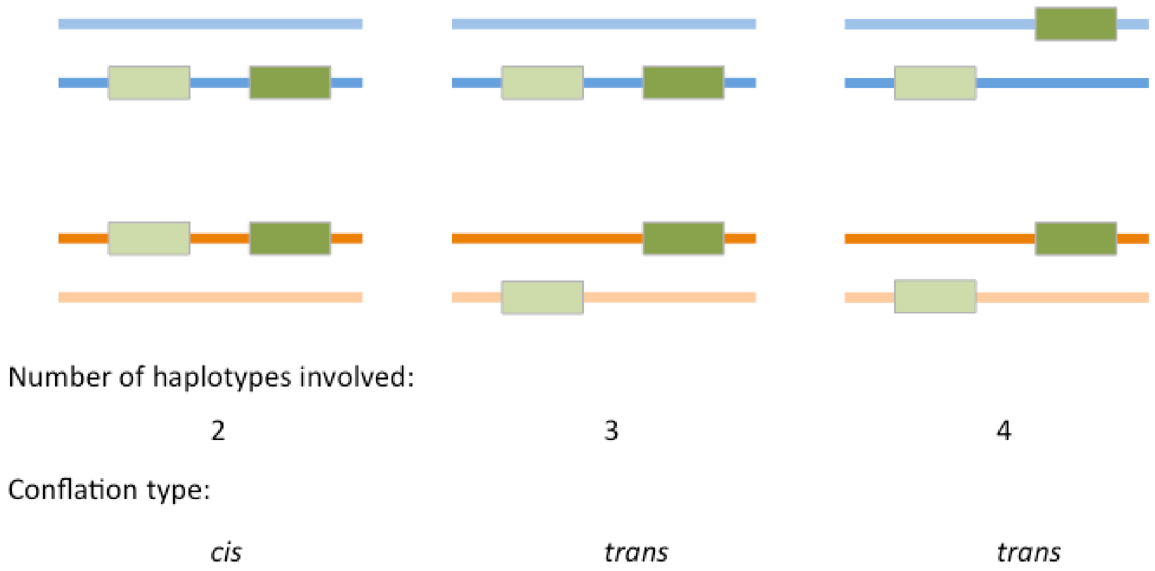

Figure S7: Illustration of *cis* vs. *trans* conflations

In each case, two shades of blue and two shades of orange colors depict the two homologous haplotypes per diploid individual. Between the two diploid individuals, conflation of two IBD segments (colored in two different shades of green) can occur in three configurations, involving either 2, 3, or 4 haplotypes. We call the scenario involving 2 haplotypes *cis*, and the scenarios involving 3 or 4 haplotypes *trans*, in which case the conflation is induced by diploidy.
