# Supplementary material for: Conflation of Short Identity-by-Descent Segments Bias Their Inferred Length Distribution
Source: G3 (Bethesda). 2016 Mar 1;6(5):1287–96. doi: 10.1534/g3.116.027581 (PMC4856080; doi:10.1534/g3.116.027581)
Supplement: Supplemental Material [file supp_g3.116.027581_TableS1.pdf]

Table S1: summary of segment age binned by length

Length bin	Number of segments	Mean age	Max age
[0.1, 0.2)	33,404,825	1134.432	2997
[0.2, 0.3)	5,760,221	754.976	2974
[0.3, 0.4)	1,513,440	611.934	2535
[0.4, 0.5)	477,898	531.359	2043
[0.5, 0.6)	166,887	502.269	1520
[0.6, 0.7)	63,098	476.703	1356
[0.7, 0.8)	25,216	454.187	1097
[0.8, 0.9)	10,314	442.023	1002
[0.9, 1.0)	4,361	417.529	1002
[1.0, 1.1)	1,967	397.696	813
[1.1, 1.2)	879	372.242	743
[1.2, 1.3)	479	339.493	698
[1.3, 1.4)	213	291.944	536
[1.4, 1.5)	138	269.775	516
[1.5, 1.6)	82	200.244	496
[1.6, 1.7)	64	126.031	506
[1.7, 1.8)	55	119.873	448
[1.8, 1.9)	33	86.970	382
[1.9, 2.0)	27	107.222	465
[2.0, 2.1)	25	75.040	182
[2.1, 2.2)	22	80.182	183
[2.2, 2.3)	19	63.947	112
[2.3, 2.4)	20	73.850	218
[2.4, 2.5)	17	53.235	109
[2.5, 2.6)	13	55.923	115
[2.6, 20)	155	40.948	129

These are summaries of ages of IBD_{ARG} segments pooled across five 20-Mb simulated regions. IBD_{ARG} segments were extracted by sampling ARGs every 0.01cM. For each bin of size 0.1cM, we then computed the number of segments, and the mean, and max ages of the segments. Note that for segments between 0.1 to 0.2cM, the maximum age is near our 3,000 generations threshold, suggesting that there are probably other segments in this length bin (and below) in the dataset that were not detected by our approach.
