# Supplementary material for: Conflation of Short Identity-by-Descent Segments Bias Their Inferred Length Distribution
Source: G3 (Bethesda). 2016 Mar 1;6(5):1287–96. doi: 10.1534/g3.116.027581 (PMC4856080; doi:10.1534/g3.116.027581)
Supplement: Supplemental Material [file supp_g3.116.027581_TableS2.pdf]

Table S2: parameter estimates of the age distributions by binned length as a function of mixture of two gamma distribution.

Length bin	λ_1	λ_2	k_1	k_2	θ_1	θ_2
[0.1, 0.2)	0.145	0.855	31.935	16.016	5.318	233.213
[0.2, 0.3)	0.303	0.697	42.327	11.369	8.027	108.881
[0.3, 0.4)	0.472	0.528	53.242	8.772	11.056	67.058
[0.4, 0.5)	0.420	0.580	110.855	3.862	14.217	42.641
[0.5, 0.6)	0.431	0.569	185.739	2.234	18.818	30.208
[0.6, 0.7)	0.638	0.362	96.634	4.433	13.333	42.125
[0.7, 0.8)	0.637	0.363	134.651	3.120	9.569	53.708
[0.8, 0.9)	0.077	0.923	2.275	195.161	50.970	8.669
[0.9, 1.0)	0.155	0.845	2.006	178.235	74.907	5.721
[1.0, 1.1)	0.161	0.839	1.792	149.474	80.832	5.229
[1.1, 1.2)	0.219	0.781	1.612	134.324	99.045	4.200
[1.2, 1.3)	0.270	0.730	1.601	89.074	117.072	3.522
[1.3, 1.4)	0.409	0.591	3.753	28.869	94.524	4.432
[1.4, 1.5)	0.465	0.535	3.466	32.213	159.563	2.551
[1.5, 1.6)	0.659	0.341	3.026	30.547	150.939	2.707
[1.6, 1.7)	0.646	0.354	5.686	10.687	2.918	83.972
[1.7, 1.8)	0.891	0.109	4.016	20.787	230.863	1.807
[1.8, 1.9)	0.954	0.046	3.220	23.367	20.424	16.284
[1.9, 2.0)	0.127	0.873	74.104	0.474	1.792	65.690
[2.0, 2.1)	0.311	0.689	22.846	1.172	9.417	10.281
[2.1, 2.2)	0.931	0.069	3.123	25.672	1041.529	0.170
[2.2, 2.3)	0.717	0.283	2.552	18.934	152.687	0.678
[2.3, 2.4)	0.848	0.152	3.173	20.154	4.212	30.693
[2.4, 2.5)	0.717	0.283	3.627	9.927	247.254	0.392
[2.5, 2.6)	0.854	0.146	3.291	16.992	12996.000	0.009
[2.6, 20)	0.819	0.181	2.206	19.722	15.941	1.844

For each binned length the age distribution is modeled as a mixture of two gamma distributions each parameterized with a shape (k_i) and scale (θ_i) parameters and weighted by λ_i. The parameters were estimated using an EM algorithm

implemented in the R package mixtools. For bins where more than 1,000 segments are available, a randomly selected subset of 1,000 segments was used for parameter estimation.
