# Supplementary material for: Conflation of Short Identity-by-Descent Segments Bias Their Inferred Length Distribution
Source: G3 (Bethesda). 2016 Mar 1;6(5):1287–96. doi: 10.1534/g3.116.027581 (PMC4856080; doi:10.1534/g3.116.027581)
Supplement: Supplemental Material [file supp_g3.116.027581_TableS3.pdf]

Table S3: mutation rate estimates using different length cut-offs.

Minimum length (cM)	Proportion conflated	Estimate of μ (x10^{-8} bp per gen)
1	0.341 (0.325, 0.356)	1.312 (1.287, 1.337)
1.5	0.130 (0.105, 0.158)	1.345 (1.220, 1.482)
2	0.073 (0.0456, 0.106)	1.279 (1.138, 1.444)
2.5	0.064 (0.0311, 0.105)	1.267 (1.093, 1.470)

The median estimates across 1,000 simulated sets are presented, with 5th and 95th percentile values given in parenthesis. The mutation rate estimates become less inflated as the threshold for minimum length increases, though the confidence interval widens due to fewer and fewer segments available for analysis. Also note that for longer segments, one could use the core regions that are unlikely to be conflated for more accurate mutation rate estimation, which is not modeled here.
